# Understanding the Mediating Effect of Child Abuse and Poor Mental Health on the Use of Adolescent Family Violence: Findings From an Australian Study

**DOI:** 10.1111/1468-4446.70022

**Published:** 2025-08-09

**Authors:** Brittany Ralph, Steven Roberts, William Lukamto, Kate Fitz‐Gibbon, Silke Meyer

**Affiliations:** ^1^ School of Law and Social Justice University of Liverpool Liverpool UK; ^2^ School of Social Sciences Monash University Melbourne Australia; ^3^ News and Media Research Centre University of Canberra Canberra Australia; ^4^ Faculty of Business and Economics at Monash University and Melbourne Law School at University of Melbourne Melbourne Australia; ^5^ School of Allied Health, Sport and Social Work at Griffith University and Monash Gender and Family Violence Prevention Centre Monash University Melbourne Australia

**Keywords:** adolescent family violence, adolescent violence in the home, child to parent violence, disability, mental health, neurodivergence

## Abstract

There is increasing recognition of the use of family violence by children and young people, and the need to build the evidence base on understanding this form of violence. Adolescent family violence (AFV, also referred to as adolescent violence in the home) refers to the use of violence by a young person against another family member within the home, and can include physical, verbal, emotional, psychological, financial and/or sexual abuse and property damage. This article presents findings from a secondary analysis of data from the *Adolescent Family Violence in Australia* (AFVA) study—the first national study of the nature, prevalence and impacts of AFV in Australia. The AFVA study involved an online survey of 5021 young people aged 16–20. Drawing from a subset of this survey data, this article aims to better understand how correlations between disability, poor mental health and use of AFV relate to young people's experiences of child abuse. The findings provide further evidence that young people's use of family violence in the home is interrelated to their own family violence victimisation during childhood. Findings presented here reiterate the need to recognise and respond to children experiencing family violence as victim‐survivors in their own right. Early and age‐appropriate child‐centred interventions would create opportunities to mitigate adverse outcomes, including poor mental health and the intergenerational transmission of violence.

## 1 Introduction

Adolescent family violence (AFV) refers to the use of violence by a young person against another family member within the home, and can include physical, verbal, emotional, psychological, financial and/or sexual abuse and property damage (Fitz‐Gibbon et al. 2022a; Royal Commission into Family Violence [RCFV] 2016). It encompasses sibling violence, adolescent violence towards parents and grandparents, and is referred to elsewhere as adolescent violence in the home (AVITH) and child to parent violence (see, inter alia, Elliott et al. 2020; Condry and Miles 2014; Campbell et al. 2020). Mirroring definitions of intimate partner violence (IPV), and specifically coercive control (see, inter alia, Stark 2007; Barlow and Walklate 2022), AFV is conceptualised as a pattern of violent or controlling behaviour that may or may not encompass physical violence (Campbell et al. 2020; Tambasco 2024). Existing literature recognises that young people predominantly articulate their use of AFV as a response to other violence occurring in the home–a crucial distinction from IPV (c.f. Fitz‐Gibbon et al. 2022a).

Prevalence estimates of AFV remain scarce. A recent Australian study found that approximately 10% of family violence incidents reported to police in Victoria involved a perpetrator aged 19 years or younger, and 7% involved a perpetrator aged 17 years or younger (Campbell 2021). Meanwhile, using an expansive definition, Fitz‐Gibbon et al. (2022a) found that some form of AFV had been perpetrated by approximately 20% of their sample of over 5000 young people living in Australia. Despite being a unique and pervasive phenomenon—and one that can have significant, long‐term impacts on affected family members (Edenborough et al. 2008; Boxall and Sabol 2021)—AFV remains under‐researched and largely invisible within criminal justice and family violence policy in Australia (Fitz‐Gibbon et al. 2018; Campbell et al. 2020) and internationally (see, inter alia, Condry and Miles 2014). As noted by Condry and Miles (2014, 259), criminologists have long ‘construct[ed] adolescents as potential offenders in the public sphere, but not within the home’.

Adding to the evidence base on this important issue, this article presents findings from a secondary analysis of data collected as part of the *Adolescent Family Violence in Australia* (AFVA) study (see Fitz‐Gibbon et al. 2022a, 2022b). That original study offers in‐depth insights into the nature, prevalence and impacts of AFV in Australia, as well as the dearth of appropriate support services for the young people impacted. The data is drawn from a comprehensive survey that was completed by 5021 young people living in Australia aged 16–20. The survey included close‐ended and free‐text questions about participants' sociodemographic characteristics, current living arrangements, and experiences of witnessing violence between other family members, being subjected to direct forms of abuse perpetrated by other family members, and their own use of violence against family members.

Among its key findings, the AFVA study found that young people with at least one form of disability were 2.8 times more likely to have used severe forms of AFV. This is concerning, given it might reinforce the pathologisation of violence as symptomatic of disability (on this, see also Renehan and Fitz‐Gibbon 2022). However, this may be related to the subsumption of ‘poor mental health’ into the broader category of ‘disability’. Poor mental health is a well‐documented outcome among children and young people growing up with victimisation experiences, including childhood experiences of family violence, and may include presentations of anxiety, depression, self‐harm and suicidal behaviours (see, inter alia, Doroudchi et al. 2023). It stands to reason, therefore, that it may occur *alongside* use of AFV (rather than being a causal factor) as a consequence of victimisation. Indeed, the main report notes that the abovementioned odds ratio reduces to 1.2 after controlling for potential ‘confounding’ factors (Fitz‐Gibbon et al. 2022a), though details of these confounding factors are not provided.

This article seeks to build on the analysis presented in the AFVA reports by examining how the correlations between disability and use of AFV relate to young people's experiences of child abuse and subsequent/consequent experiences of ‘poor mental health’. This article is broadly structured into five key sections. We begin by reviewing existing research examining AFV, highlighting the crucial contribution of the AFVA study as well as the need for further investigation into the intersection between disability and the use of AFV identified in this national dataset. We then outline the study design, including the secondary mixed‐methods analyses that inform the results presented here. First, our qualitative findings indicate that—because poor mental health and the use of AFV often follow from prior victimisation—omitting childhood abuse may inflate the observed association between disability and AFV use. Next, we used a set of multivariable linear probability models, estimated via Stata's paramed command, to decompose the total effect of disability on AFV use into direct and indirect (mediated by childhood abuse) components. In doing so, this article further demonstrates that young people's use of family violence is deeply intertwined with their own experiences of family violence victimisation during childhood. Our discussion and conclusion build on existing calls to recognise children and young people affected by family violence first and foremost as victim–survivors in their own right.

## 2 Adolescent Family Violence: The State of Knowledge

An emerging body of research examines the prevalence and nature of AFV in Australia and internationally. Key findings in this area suggest that: the best predictor for use of violence against a parent is ‘the presence of similar behaviour in other contexts’ (see review by Simmons et al. 2018); mothers are the most common primary target of AFV, and this violence tends to be frequent, ongoing, start earlier in the adolescent's life and result in significant impacts/harm (Boxall and Sabol 2021); and—of particular relevance here—that experiences of child abuse or other adverse childhood events are often present prior to an adolescents' use of violence in the home (Campbell et al. 2020, 2023; Renehan and Fitz‐Gibbon 2022). Researchers have observed that violence is used as a form of retaliation or self‐defence, in that young people are responding to actual or anticipated abuse; or where the use of violence is predicated on social learning, in that young people using AFV copy and ‘“replay” the violence and controlling behaviour which they have seen exhibited by adults in their lives’ (Campbell 2021, 97; see also Fitz‐Gibbon et al. 2022a). Research undertaken with Australian practitioners has also evidenced the trend whereby young people begin to use severe violence against their mother or siblings soon after escaping their violent father, as well as instances where an adolescent is coerced or coached by their father to use violence (Campbell 2021; Douglas and Walsh 2018; Renehan and Fitz‐Gibbon 2022). Beyond these explicit forms of intergenerational transmission, researchers have examined how experiencing FV between parents or carers negatively impacts a child's cognitive development (Carlson 2000) and ability to develop appropriate attachments to their parents (as well as others) (Holt 2013), and how a lack of parental warmth can lead to narcissism and rejection schemas (Calvete et al. 2015), all of which were found to increase the likelihood that a young person will use violence against a family member. Structural perspectives examine factors beyond the family unit, in particular the role of gender norms. For example, Cottrell and Monk (2004) assert that the societal devaluing of motherhood and social norms surrounding men's entitlement to power and control are key drivers of AFV.

Taken together, this research illustrates the unique nature of AFV and the multiple, intersecting factors that underpin it. Through a comprehensive and widely disseminated survey (*n* = 5021), the AFVA study builds on this growing body of literature by offering in‐depth data about all forms of AFV from the perspective of the young people using it, and the kinds of support these young people found helpful (further details of the study's methodology are provided below). The study's findings are presented across two reports (Fitz‐Gibbon et al. 2022a, 2022b). To briefly summarise:

- One in five respondents had used some form of violence against a family member at least once (20%, *n* = 1006), a larger proportion of those being respondents assigned female at birth^1^ (*n* = 762).
- The most common forms of violence reported were verbal abuse (15%, *n* = 734), physical violence (10%, *n* = 490) and emotional/psychological abuse (5%, *n* = 245).
- On average, adolescents began using violent behaviours at age 11 (SD = 3.9) and it was more often episodic (less than once a month, *n* = 553) than regular (at least once a month, *n* = 453).
- There was a significant correlation between victimisation and use of AFV, with 89% (*n* = 896) of adolescents who used family violence reporting they had experienced child abuse, including targeted abuse and/or experiences of family violence between other family members. Qualitative data suggest this violence was often retaliatory, self‐defence or a result of social learning.
- Young people with a disability were 2.8 times more likely to use severe forms of AFV in the home than young people who did not have a disability.

Adolescents who use family violence are not a homogenous cohort—their unique experiences and intersecting forms of disadvantage shape the nature, severity and frequency of their use of violence. However, as noted here, initial analysis of the quantitative dataset suggests that young people who report living with a disability are among the most likely to use some form of AFV.

## 3 The Relationship Between Disability, Mental Ill‐Health and the Use of AFV

Many studies report a link between disability and use of AFV, particularly in relation to mental ill‐health and neurodivergence. In a thorough review of literature pertaining to child‐to‐parent abuse, Simmons et al. (2018) note the higher incidence of depressive symptomology (e.g., Ibabe et al. 2014), reported suicide attempts and self‐harm (e.g., Kennedy et al. 2010), Attention Deficit Hyperactivity Disorder (ADHD), Conduct Disorder and Oppositional Defiant Disorder among young people using violence in the home (e.g., Contreras and Cano 2014). In the Australian context, Campbell et al. (2020) found that 47.4% of a random sample of Victorian police files related to AFV occurrences in 2017 featured adolescents with a disability, with 24% featuring adolescents with Autism Spectrum Disorder. However, the relationship between mental ill‐health, disability and aggression is debated.

A key disclaimer across this literature, is that these findings are not only correlational (rather than causal), but also potentially tautological, given that the use of violence in the home could justify diagnoses associated with disordered patterns of behaviour (Simmons et al. 2018). Likewise, Sutherland et al. (2022, 4) note the importance of understanding disability as a ‘contextual’ factor, rather than a ‘risk’ factor, as it can often ‘co‐occur with violence but the pathways that connect them have not yet been established’. Likewise, practitioners warn against the medicalisation of family violence as symptomatic of neuro‐divergence, noting that while biological factors may play a part, so too does trauma and childhood neglect (Renehan and Fitz‐Gibbon 2022). Further complicating this discussion Sutherland et al. (2022) note that the tendency to apply adult IPV frameworks that define violence as an attempt ‘physically or psychologically dominate, coerce and control others in their family’ (Howard and Rottem 2008, 10) implies an assumed intentionality that may not be present among young people with disability. Indeed, much literature pertaining to disability and family violence addresses the far greater risk of victimisation, rather than a greater likelihood of using violence (see, e.g., Breiding and Armour 2015; Powers et al. 2009).

While the relationship between disability and use of violence identified in the AFVA study appears to align with these broader trends, it is thus vital to more precisely examine the nature of this link to avoid pathologising AFV as a symptom of disability and further stigmatising young people with disability. To this end, we conducted an in‐depth, secondary analysis of the AFVA data including further qualitative analysis of participants' open‐ended responses and a targeted set of statistical tests.

## 4 Research Design

This article presents findings from a subset of data taken from the *Adolescent Family Violence in Australia* (AFVA) survey data set. AFVA is a recently completed national project which involved an online survey of 5021 young people aged 16–20 to examine the nature, prevalence and impacts of AFV in Australia. Utilising the AFVA national dataset, the secondary analysis presented here sought to test the following hypothesis:

Experiencing child abuse acts as a mediator, such that it explains—and if omitted would inflate—the effect of disability on the likelihood of using AFV.

The specific methods are set out in detail below. The term ‘cognitive disability’ is used in this paper as a category that encompasses neuro‐divergence (e.g. autism spectrum disorder and attention deficit hyperactivity disorder), intellectual disability and/or impairment, and learning disability, however this category is referred to as ‘mental disability’ within the statistical models. Further, the term ‘poor mental health’ is based on young people's self‐reports in the survey that they were living with ‘poor mental health affecting day to day functioning’ rather than any specific mental health conditions or diagnoses.

## 5 Sample

Survey respondents were recruited through online research panels managed by Open Research Unit (ORU). The survey was sent to members of the ORU's online research panels who were aged 16–20 at the time of data collection. Overall, 5021 young people completed the survey (see Table 1 for key summary statistics of key variables). Respondents aged 16 or 17 years old required the consent of their primary guardian to be included in the ORU panels. Two thirds of the sample identified that they were assigned female at birth (*n* = 3348), and one third identified they were assigned male at birth (*n* = 1623). This sample was recruited using non‐probability protocols and the survey was only provided in English, meaning it is not representative of the broader Australian population. As such, the findings presented here are not generalisable and may not be replicated if the survey is administered again using a probability sample.

**TABLE 1:** Summary statistics of the variables.

Variable	Freq.	Percent	Cum.	Min	Max
Use of any forms of violence	5021	100.00		0	1
No	4015	79.96	79.96		
Yes	1006	20.04	100.00		
At least one disability	1748	36.45	100.00		
Disability: Poor mental health	4796	100.00		0	1
No	3738	77.94	77.94		
Yes	1058	22.06	100.00		
Sex	4971	100.00		0	1
Male	1623	32.65	32.65		
Female	3348	67.35	100.00		
Gender normativity	4988	100.00		1	2
Normative	4773	95.69	95.69		
Non‐normative	215	4.31	100.00		
Culture	5015	100.00		1	2
Speak English most of the time at home	4490	89.53	89.53		
Speak a language other than English at home	525	10.47	100.00		
Aboriginal and torres strait islander status	5018	100.00		0	2
Non‐indigenous	4723	94.12	94.12		
ATSI	256	5.10	99.22		
Prefer not to say	39	0.78	100.00		
Education	5016	100.00		1	8
Primary school	166	3.31	3.31		
Year 7	2	0.04	3.35		
Year 8–9	17	0.34	3.69		
Year 10	656	13.08	16.77		
Year 11	1058	21.09	37.86		
Year 12	2569	51.22	89.07		
Tafe, certificate or diploma	534	10.65	99.72		
Undergraduate	14	0.28	100.00		
Country of birth	5021	100.00		1	13
Australia	4221	84.07	84.07		
England	88	1.75	85.82		
China	51	1.02	86.84		
India	92	1.83	88.67		
New Zealand	70	1.39	90.06		
Philippines	39	0.78	90.84		
Vietnam	33	0.66	91.50		
South Africa	28	0.56	92.05		
Italy	6	0.12	92.17		
Malaysia	45	0.90	93.07		
Sri Lanka	21	0.42	93.49		
Other (please specify)	294	5.86	99.34		
Prefer not to say	33	0.66	100.00		
Victimisation of any form of violence	5021	100.00		0	1
No	3507	69.85	69.85		
Yes	1514	30.15	100.00		

Throughout this article, when reporting from the qualitative data set, survey respondents are referred to by their demographic information including:

- Gender identity
- Age
- Sexuality
- Place of birth (and language(s) spoken at home other than English)
- Disability status (PMH = poor mental health, ASD = Autism Spectrum Disorder, ADHD = Attention Deficit Hyperactivity Disorder, LD = learning disability, VI = visual impairment, ND = no disability)

## 6 Data Collection Procedures

The survey data collection was conducted between September and October 2021 using Qualtrics Software. Survey respondents were asked a series of close‐ended and free‐text questions about their sociodemographic characteristics, their current living arrangements, and their experiences of:

- witnessing violence between other family members,
- being subjected to direct forms of abuse perpetrated by other family members, and
- their use of violence against other family members.

Respondents who self‐reported having experienced, witnessed and/or used violence were asked to provide detailed information about: the nature of their experiences and use of violence (including age of onset, frequency of behaviours and relationship to the perpetrator/victim); the impacts of their experiences and use of violence in a range of domains; and the kinds of services and supports they had found most and least helpful and why.

In this study, the term ‘family member’ encompassed biological parents, adoptive parents, step‐parents and foster carers, siblings, grandparents, extended family members (e.g. aunts, uncles and cousins), chosen family members and Aboriginal and Torres Strait Islander kinship relationships. As noted above, ‘violence’ included physical violence, property damage, verbal abuse, emotional/psychological abuse, sexual abuse and threatening behaviours. In this article, both ‘witnessing violence between other family members’ and ‘being subjected to violence to direct forms of abuse’ are referred to as ‘experiencing child abuse’, in line with national and international policy developments (Department of Social Services 2022; Haslam et al. 2023; Meyer 2018) given the severe impacts of both.

## 7 Qualitative Analysis

This paper draws on a mixed methods design combining quantitative and qualitative strands to explore adolescent family violence. While the quantitative analysis was informed by a post‐positivist framework to identify statistical associations, the qualitative strand followed a constructivist epistemological approach. In this sense, we adopt a pragmatist approach that prioritises the practical usefulness of combining multiple methods over the need to adhere to a single epistemological framework (for a contemporary outline of this position, see Hothersall 2019).

Qualitative data for this article was extracted only from respondents who reported using some form of family violence at least once and having at least one disability. The analysis was initially aimed at examining survey respondents' ‘reasoning’ for their use of violence, which was gathered from their responses to the following open‐ended questions:

Q35. Were there particular factors or circumstances that occurred in the immediate lead up to those behaviours? (Yes, Please describe)

Q41. What do you believe are the factors, if any, that led to your use of violence?

Approximately one‐quarter of participants in this sub‐sample answered ‘no’ to this question and did not provide an answer to the subsequent open‐ended question. Among those who answered ‘yes’, a very large portion provided one‐word or half‐sentence responses. Nonetheless a clear data set emerged from here that prompted the research team to analyse qualitative data pertaining to the emotional impacts participants had experienced as a result of the family violence in their home (either experienced, witnessed or used). This data was captured in responses to the following question:

Q38. Please describe the emotional impact/consequences.

This open‐ended question was asked at the end of the survey, and as such was not grouped with the sets of questions relating specifically to experiencing, witnessing or using family violence, meaning survey respondents could detail the impacts of any/either form of family violence.

All data was extracted, entered into NVivo qualitative analysis software and coded thematically to draw out patterns in the dataset (Bryman 2016). Themes were developed iteratively through an inductive, data‐driven process, supported by regular team discussions to account for researcher positionality, address potential bias and enhance the academic rigour of the analysis. Following Braun and Clarke's (2006) approach, initial coding was conducted by the first author, at which point a set of clear themes were generated including ‘self‐defence’, ‘mental health’ and ‘stress’, which neatly reflected existing literature. Given the aims of the analysis and brevity of each piece of data, further theoretical coding was not necessary. The second author then cross‐coded a subset (circa 25%) of data to establish inter‐rater reliability, and finally key themes were presented to the broader research team for discussion and consolidation.

## 8 Quantitative Analysis

**FIGURE 1:**
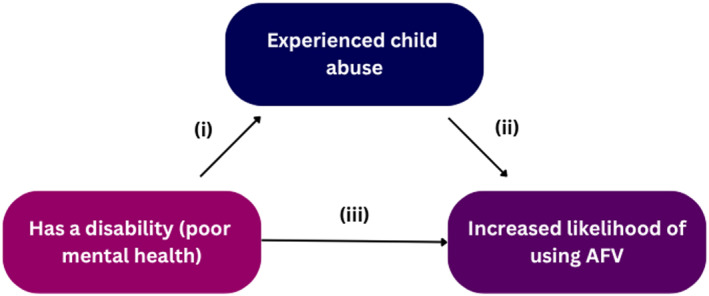
Mediation model.

Mediation analysis was performed in order to test the hypothesis that experiencing child abuse mediated the effect of having a disability on the likelihood of using AFV. Of the four categories of disability measured by the survey (physical disability, visual/hearing disability, poor mental health and cognitive disability), poor mental health was chosen as the independent variable. This decision was based on the results of our qualitative analysis, which suggested that poor mental health may be a consequence of child abuse, and (particularly given the cross‐sectional nature of the survey) may have been contributing to an overstated effect of disability on the likelihood of using of AFV. The increased likelihood of using AFV was chosen as the dependent variable, and having experienced FV was set as the mediator as shown in Figure 1. Per this model, pathways (i) and (ii) will investigate the indirect effect poor mental health has on increased likelihood of using AFV if one has experienced child abuse. Pathway (iii) will investigate the direct effect poor mental health has on increased likelihood of using AFV. In this way, we determined if experiencing child abuse fully or partially mediates the relationship between disability and increased likelihood of using AFV.

Mediation analysis was performed using SPSS via the ‘paramed’ command (without interaction^2^). This allowed us to look at the controlled direct (CDE) and indirect effects (NIE) of the relationships. To increase internal validity and mirror the AFVA study for more accurate comparisons, we will also be relying on ‘sex’ (assigned at birth), ‘culture’, ‘education’ and ‘country of birth’ as control variables. As such, we will be relying on the controlled direct effects and the natural indirect effects to determine if full or partial mediation exists in the model.

## 9 Dependent Variable

### 9.1 Use of Any Forms of Violence (perp_ANY)

This binary variable takes into consideration all forms of family violence measured in the ORU survey including physical violence (e.g. hitting, slapping, pushing, punching or kicking), property damage (e.g. intentionally destroying someone's property or belongings as intimidation), verbal abuse (e.g. yelling, swearing), threats to harm, threats to kill, threats to harm someone close (e.g. a pet or friend), sexual abuse, non‐fatal strangulation, sexual identity‐based abuse (e.g. exclusion from family due to sexual identity), gender identity‐based abuse (e.g. discrimination and prejudice due to gender identity) and any other forms of abuse the respondent self‐reports. 0 = Respondent did not report using any of the above forms of family violence, 1 = Respondent reported using at least one of the above forms of family violence.

## 10 Independent Variable

### 10.1 Poor Mental Health (disability_MI)

This binary variable takes into consideration poor mental health affecting day to day functioning as measured in the survey. 0 = Respondent did not report suffering from poor mental health, 1 = Respondent reported suffering from poor mental health. This was chosen as our independent variable as it was the largest disability group in proportion in our sample and had the greatest effect on the use of AFV in a previous study.

## 11 Mediation Variable

### 11.1 Victimisation of Any Form of Violence (target_ANY)

This binary variable records whether the respondent has experienced any of the forms of family violence measured in the ORU survey including physical violence (e.g. hitting, slapping, pushing, punching or kicking), property damage (e.g., intentionally destroying someone's property or belongings as intimidation), verbal abuse (e.g., yelling, swearing), threats to harm, threats to kill, threats to harm someone close (e.g., a pet or friend), sexual abuse, non‐fatal strangulation, sexual identity‐based abuse, gender identity‐based abuse, and any other forms of abuse the respondent recognises. 0 = Respondent did not report experiencing any of the above forms of family violence, 1 = Respondent reported experiencing at least one of the above forms of family violence.

## 12 Control Variables

### 12.1 Sex (Sex)

This binary variable records the respondent's biological sex assigned at birth. 0 = male, 1 = female.

### 12.2 Gender Normativity (GENDER_cat2)

This binary variable records the respondent's gender normativity (i.e. *cis*‐gendered vs. gender non‐normative). 1 = Normative, 2 = Non‐normative.

### 12.3 Culture (NESB)

This is a binary variable measuring if the respondent speaks English at home most of the time or not. 1 = Speaks English most of the time at home, 0 = Speaks a language other than English at home.

### 12.4 Aboriginal and Torres Strait Islander (ATSI)

This binary variable records whether the respondent identifies as Aboriginal and Torres Strait Islander. 0 = Non‐Indigenous, 1 = Identified as Aboriginal and/or Torres Strait Islander.

### 12.5 Education (Education)

This is an ordinal variable measuring the level of education the respondent held. 1 = Primary school, 8 = Undergraduate or above.

### 12.6 Country of Birth (COB2)

This is a nominal variable indicating the respondent's country of birth. The countries used as options for this variable include: England, China, India, New Zealand, Philippines, Vietnam, South Africa, Italy, Malaysia and Sri Lanka. All other countries were included in the “Other (please specify)” category.

## 13 Results

### 13.1 Qualitative Insights

At the outset of this project, we analysed the qualitative data to gain a deeper understanding of how respondents who reported having at least one disability made sense of their use of family violence. Of those who chose to include a short answer, many were relatively simple, with respondents commenting, ‘anger issues’ (Female, 18, Heterosexual, AUS [Mandarin spoken at home], PMH), ‘disputes’ (Female, 18, Heterosexual, AUS, PMH) or ‘Arguments and drugs’ (Female, 16, Heterosexual, AUS [Samoan spoken at home], PMH). In other (albeit very rare) instances, the respondents attributed their use of violence solely to a dissatisfaction with the behaviour of other members of their family. One respondent commented, ‘my brother was being extremely disrespectful to me leading up to this’ (Male, 20, Heterosexual, AUS, PMH), while another who had reported engaging in strangulation commented ‘my mum was going to reveal secrets I told her to somebody’ (Male, 20, Heterosexual, AUS, PMH/ASD). However, the vast majority of respondents who provided qualitative responses attributed their use of violence to self‐defence/retaliation, stress, and/or mental health/disability related episodes.

In line with the literature reviewed above (e.g., Campbell 2021), self‐defence and social learning emerged as two of the most common drivers. In relation to the former, respondents frequently attributed their use of violence to a need for self‐protection. For example, one respondent wrote, ‘They abused me first and I was only defending myself’ (Female, 19, Heterosexual, AUS, LD/PMH), while another shared, ‘My mum hit me first, acted aggressively, and backed me into a corner’ (Demi girl, 18, Lesbian, AUS, PMH). These statements reflect a broader pattern in the data in which violence was clearly framed as a reactive, rather than pre‐emptive, behaviour. Separate but relatedly, participants cited the normalisation of violence in the home—most often by parents—reflecting processes of social learning (Campbell 2021). Illustrating this viewpoint, one respondent shared, ‘…being abused from a young age, led to mentality when angry that violence is acceptable’ (Male, 17, Gay, AUS, ASD). Similarly, another young person surveyed stated they, ‘would use violence because I saw that as the way to show that you are stronger than the other person, which I learned from my father’ (Demi boy, 18, Pansexual/Demisexual/Demiromantic, AUS, PMH). There was also a strong related theme whereby respondents viewed their use of violence as a reaction to long periods of emotional abuse. As these young people explained:

Being constantly put down and told I was worthless, so I would get defensive and stand up for myself

(Female, 18, Heterosexual, AUS, PMH)

Being hit and belittled all the time, not knowing how to get my emotions out without yelling, having to hit back to make them stop hurting me.

(Female, 20, Bisexual, AUS, PMH/ADHD/ASD/VI)

As well as highlighting the use of violence as a form of self‐defence or retaliation, these comments illustrate the sustained and significant forms of abuse that respondents who reported using violence and having a disability had been subject to during their childhood. Notably, too, it seems feasible that these experiences were a significant contributor to the poor mental health these respondents report—a point we return to in later sections of this article.

Beyond victimisation, many respondents explicitly attributed their use of violence to mental illness, cognitive disability or neurodivergence related symptoms or episodes, particularly when their unique needs were not being met. This viewpoint was expressed particularly explicitly by female‐identifying respondents:

undetected mental health disorders including adhd, ptsd and bpd. I'm not excusing use of aggression/violence, but having these diagnosed in my earlier childhood would have prevented a lot.

(Female, 20, Heterosexual, AUS [Greek spoken at home], PMH/ADHD/LD)

My environment; what I saw/learnt, my absolute need to defend myself, and my undiagnosed and ignored behavioural issues, in which I was punished for. Retaliation.

(Female, 17, Heterosexual, AUS, PMH/ADHD/ASD/LD)

Severe untreated mental health/undiscovered neurodivergency, and homesickness after immigration

(Female, 18, Pansexual, England, ADHD/PMH)

Within this theme, male‐identifying survey respondents commented:

poor anger management due to mental health

(Male, 20, Pansexual, AUS [German spoken home], ASD/PMH/PI)

Being yelled at by dad, sister being incredibly irritating regardless, possibly my aspergers‐ never could handle pressure/annoyances very well.

(Male, 20, Heterosexual, Greece, ASD/Other: ‘Obese’)

As illustrated in these excerpts, there were gendered patterns in how respondents reflected on the role of mental health or cognitive disability in their use of AFV. While it is beyond the scope of this article, it is worth noting here and will be explored in more depth in a separate paper. Crucial, here, is that the respondents often cited the untreated nature of their condition (or worse still, the sense that they were punished for it) as a trigger for their use of violence, rather than it being inherent to the condition itself. This aligns with research cautioning against pathologising young people's behaviour without accounting for trauma, unmet support needs, and the sociocultural context in which neurodivergence is experienced (Botha and Frost 2020; Goodley 2013).

Additionally, in line with the concept of ‘minority stress’, young people with mental illness, disability or neurodivergence tend to experience greater levels of stress in their day to day lives due to ableist and stigmatising social systems and institutions (Botha and Frost 2020; see also Lund 2021). This was apparent among the experiences of young people in this study, with respondents emphasising the role of stress in their use of violence by, in some cases, stating quite plainly, ‘i was very stressed in high school, and lashed out’. (Non‐binary, 19, Queer, AUS, ASD/PMH). Others listed stress among a range of factors, with one respondent stating, ‘arguments with my mother, also exacerbated by mental ill health, life stressors’ (Non‐binary, 19, Gay, AUS, PMH), and another, ‘Stress factors, unaddressed mental health issues, counteracting abuse’ (Female, 18, Bisexual, AUS [Spanish spoken at home], ADHD/PMH).

Notably, relatively few respondents attributed their use of violence *solely* to the symptoms of their condition, instead highlighting the compounding effect of increased stress and, again, their experiences of family violence within the home. As such, while their psychosocial condition may appear to be *an* explanatory factor, we join Renehan and Fitz‐Gibbon (2022, 5) in emphasising that ‘domestic abuse and the drivers of violence should not be medicalised as autism and/or ADHD’, or indeed any form of mental illness or disability.

There is, however, an additional layer of complexity to consider; that the ‘poor mental health’ reported by participants may not simply be *a contributing factor to*, but *a consequence of their victimisation* much like, their use of violence. Indeed, when asked to reflect on the impact of family violence being present in the home, numerous respondents highlighted the immense consequences to their mental health, even those that did not report having ‘poor mental health’ in the survey item. As captured in the following comments made by four survey respondents:

Emotional outbursts and mental health problems caused by the abuse I suffered

(Female, 19, Lesbian, AUS, PMH/ASD)

I developed many mental health disorders that I deal with to this day… My father said he was under stress, I don't believe him. I don't believe he ever cared for me. I believe he just wanted power because he couldn’t get that outside of abuse

(Female, 17, Bisexual, AUS, PMH)

All the emotional neglect and abuse caused me to have severe depression, numbness and other horrible thoughts.

(Female, 17, Heterosexual, AUS [Farsi spoken at home], ND)

severe depression problems and feelings of worthlessness… inability to socialise as *i* was made shy by being physically abused for misbehaving

(Female, 19, Heterosexual, AUS, ND)

**FIGURE 2:**
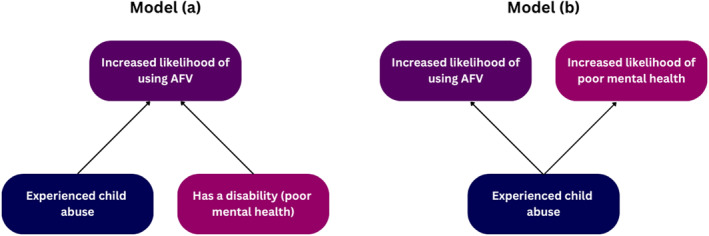
Relationship between child abuse, poor mental health and use of AFV.

Arguably, then, poor mental health and victimisation status should not be thought of as separate variables that in combination increase the likelihood of use of family violence (see Figure 2 model a). Rather, both poor mental health and use of AFV may be a consequence of victimisation (see Figure 2 model b), and this may lead to an overestimation of the correlation between poor mental health (presence of a disability) and a young person's use of family violence. Put simply, increased likelihood of use of AFV among respondents with poor mental health (a disability) may be associated with their increased likelihood of having experienced child abuse. To explore this possibility quantitatively, we developed and tested the hypothesis that: experiencing child abuse acts as a mediator, such that it explains—and if omitted would inflate—the effect of disability on the likelihood of using AFV.

### 13.2 Quantitative Insights

While cross‐sectional survey data are thought to offer limited insight into the ‘arrow of causality’ between variables, mediation analysis of this dataset does offer important insight into the relationship between poor mental health, experiences of child abuse, and use of AFV (see Tables 2, 3, 4). In this section we present these results.

**TABLE 2:** Regression results predicting use of any forms of violence (*N* = 4724; F(84,715) = 138.44, *R* ^2^ = 0.1902, Adj. *R* ^2^ = 0.1888, RMSE = 0.3626).

Predictor	*B*	SE	*t*	*p*	95% CI lower	95% CI upper
Poor mental health	0.0461	0.0136	3.38	0.001	0.0194	0.0729
Victimisation of violence	0.3637	0.0120	30.28	< 0.001	0.3402	0.3873
Sex	0.0091	0.0118	0.77	0.442	*−*0.0140	0.0321
Gender normativity	−0.0015	0.0288	−0.05	0.959	−0.0580	0.0550
Culture (NESB)	−0.0308	0.0176	−1.75	0.081	−0.0654	0.0038
ATSI	0.0011	0.0008	1.38	0.167	−0.0004	0.0026
Education	−0.0006	0.0045	−0.15	0.885	−0.0094	0.0081
Country of birth	−0.0002	0.0018	−0.12	0.905	−0.0037	0.0033
Constant	0.1152	0.0440	2.62	0.009	0.0289	0.2014

**TABLE 3:** Regression results predicting *victimisation of violence* (*N* = 4724; F(74,716) = 67.38, *R* ^2^ = 0.0909, Adj. *R* ^2^ = 0.0896, RMSE = 0.4395).

Predictor	*B*	SE	*t*	*p*	95% CI lower	95% CI upper
Poor mental health	0.2449	0.0161	15.17	< 0.001	0.2132	0.2765
Sex	0.1109	0.0142	7.83	< 0.001	0.0831	0.1387
Gender normativity	0.1822	0.0348	5.23	< 0.001	0.1139	0.2505
Culture (NESB)	−0.0324	0.0214	−1.51	0.130	−0.0743	0.0095
ATSI	0.0016	0.0009	1.71	0.087	−0.0002	0.0034
Education	0.0185	0.0054	3.42	0.001	0.0079	0.0291
Country of birth	0.0037	0.0022	1.72	0.086	−0.0005	0.0080
Constant	−0.0855	0.0533	−1.60	0.109	−0.1901	0.0190

**TABLE 4:** Mediation analysis results (bias‐corrected bootstrap with 1000 replications).

Effects					
Parameter	Estimate	Bias	SE	95% CI Lower	95% CI Upper
CDE	0.0461	0.0004	0.0159	0.0160	0.0764
NIE	0.0891	−0.0004	0.0075	0.0756	0.1051
TE	0.1352	0.0000	0.0166	0.1034	0.1660

## 14 Tables 2–4: Mediation Analysis Results

Our analysis controlled for sociodemographic variables (sex, gender normativity, culture, ATSI status, education, and country of birth). Notably, both sex and non‐normative gender identity emerged as strong predictors of victimisation, but had no direct association with perpetration when poor mental health and victimisation were considered. In the victimisation regression, being female and having a non‐normative gender identity were both associated with a higher probability of having experienced family violence during childhood (*β* = 0.1109, *p* < 0.001; *β* = 0.1822, *p* < 0.001). By contrast, neither sex (*β* = 0.0091, *p* = 0.442) nor gender normativity (*β* = −0.0015, *p* = 0.959) significantly predicted perpetration of violence in the full mediation model. This pattern suggests that gender‐based disparities in perpetration are largely explained by disparities in victimisation (and downstream mental‐health impacts), rather than by gender per se. However, investigations into the indirect gender effects on perpetration is beyond the scope of this thesis. The mediation analysis produced four key findings consistent with our hypothesised pathways (see Figure 1).

First, there is a direct effect of poor mental health on likelihood of having used AFV. Individuals who had poor mental health were more likely to also report using family violence (*β* = 0.0461, *p* = 0.001). This direct effect (CDE) accounted for 34.1% ($\frac{CDE}{TE}=\frac{0.0461}{0.1352}=0.3410$) of the total effect (TE) of poor mental health on use of AFV. This represents pathway (iii) in Figure 1 and is, as expected, similar to the results of the previous study, statistically significant at *p* < 0.01.

Second, experiencing childhood abuse directly increases the likelihood of AFV. Individuals who experienced childhood abuse prior to 18 years old were significantly more likely to use family violence (*β* = 0.3637, *p* < 0.001). This shows that a positive statistically significant relationship exists in pathway (ii) of Figure 1.

Third, individuals who had poor mental health are also more likely to have experienced childhood abuse prior to 18 years of age. Poor mental health had a large and positive statistically significant effect on predicting childhood abuse (*β* = 0.2449, *p* < 0.001). This shows a statistically significant relationship exists in pathway (i) of Figure 1.

Finally, and most crucially, poor mental health indirectly increases AFV when mediated through childhood abuse (NIE = 0.0891, *p* = 0.001). This indirect pathway explains 65.9% of the total effect ($\frac{NIE}{TE}=\frac{0.0891}{0.1352}=0.6590$), suggesting that childhood abuse plays a larger mediating role than when solely looking at direct effects between poor mental health and likelihood of AFV. This shows that a positive relationship does exist through pathways (i) and (ii) as illustrated in Figure 1. That is, having experienced child abuse partially mediates the relationship between poor mental health and use of AFV.

This confirms that the effect of poor mental health on use of AFV shows mediation effects through the variable ‘victimisation of any form of violence’. Our NIE is stronger than our CDE, suggesting that the indirect effect of poor mental health (prior to age 18)—mediated through childhood abuse—on the likelihood of a young person using AFV is stronger than the direct effect without mediation.^3^ While cross‐sectional data cannot establish temporal precedence, these results align with our prior work suggesting bidirectional links between poor mental health and abuse.

## 15 Discussion and Conclusion

Results from the AFVA study indicate that survey respondents with a disability were more likely to report having used AFV (Fitz‐Gibbon et al. 2022a). Our findings build on this by offering crucial insights into the complex relationship between disability and young people's use of violence against their family members. The qualitative analysis illustrates how respondents with cognitive disability and/or poor mental health made sense of their use of family violence in the home. In line with existing literature, self‐defence and social learning were the most commonly cited reasons for using family violence (Campbell 2021), as was the view that their violence was a reaction to long periods of emotional abuse. Rather than pathologising AFV as symptomatic of cognitive disability, neurodivergence and/or poor mental health, this data offers richer insights into how, in the context of abuse and neglect, young people with these conditions often feel they have no option but to retaliate, illustrating the cycle of regret, shame and anger this can create. Relatedly, analysis of the emotional impacts listed by survey respondents highlights the immense toll that family violence (whether experienced or used) has on the mental health of young people. This data raises further questions about the nature of the relationship between disability and use of AFV, and led to the development of our main hypothesis. Rather than victimisation and disability representing two separate variables that in combination increase the likelihood of use of AFV, we hypothesised that both poor mental health and use of AFV are a consequence of child abuse victimisation. Given the cross‐sectional nature of the survey, we surmised that this had led to an overestimation of the correlation between disability and use of AFV.

Results from the mediation analysis detailed above largely supported this hypothesis. That is, individuals who reported experiencing ‘poor mental health’ were more likely to report using family violence (CDE = 0.0461); individuals who had been subjected to child abuse prior to the age of 18 were more likely to also report using family violence (*β* = 0.3637); and most crucially, being a victim of child abuse had a positive and statistically significant indirect effect (NIE = 0.0891) on the use of AFV when mediated for poor mental health. This shows that a positive relationship does exist through pathways (i) and (ii) as illustrated in Figure 1, meaning that reporting experiences of child abuse partially mediates the relationship between poor mental health and use of AFV. In addition to this, our findings indicate the indirect effect of having poor mental health (mediated by experience of childhood abuse) on the likelihood of using AFV is stronger than the direct effect without mediation (34.1% vs. 65.9%).

These findings suggest that the relationship between experiencing child abuse, having a disability and using AFV may in many cases more closely reflect model (b) rather than model (a). That is, for some young people having poor mental health may not be a causal factor of use of AFV, but instead both might be a consequence of having experienced child abuse. While it is not possible, within the scope of this study, to define the arrow of causality between disability and use of AFV, this analysis substantiates existing calls to avoid pathologising AFV as symptomatic of disability and/or poor mental health (see also Renehan and Fitz‐Gibbon 2022). These findings particularly resonate in the current policy context in Australia where the recently released 10‐year National Plan to end Violence against Women and Children 2022–2032 acknowledges the need to build responses to children and young people as victim‐survivors in their own right, as one part of the political commitment to end gender‐based violence in one generation (Department of Social Services 2022).

Results from the AFVA study (Fitz‐Gibbon et al. 2022a, 2022b) draw crucial attention to the prevalence of AFV in Australia, as well as young people's support needs. However, as reinforced by the secondary analysis undertaken for this article, young people's use of family violence in the home is often significantly associated with their own victimisation experiences. Understanding the relationship between particular demographic characteristics and use of family violence is central to preventing it. With respect to preventing AFV, specifically, the more important factors appear to be that (a) young people are not experiencing family violence in the first place, (b) if they are, there are effective whole‐of‐family and child‐centred interventions, support and recovery services available to mitigate the effects such experiences have on their mental health and ability to regulate emotion, and (c) if they do use AFV, that young people are treated first and foremost as potential victim‐survivors in their own right. These findings support broader calls for more therapeutic, trauma‐informed approaches to accountability work with young people using family violence (Campbell 2021; Campbell et al. 2020; Department of Social Services 2022; Fitz‐Gibbon et al. 2022b). Finally, the data limitations raised in this article highlight the need for future research to capture children and young people's experiences and use of violence in the home longitudinally, to establish temporal order of victimisation experiences and adverse outcomes, including poor mental health and the intergenerational transmission of violence.

These findings carry significant implications for both policy and practice. They reaffirm the need for early intervention and crisis response systems that recognise children and young people as victim‐survivors in their own right. School‐based supports, specialist domestic and family violence services, mental health services, and youth justice responses should adopt trauma‐informed approaches that acknowledge the cumulative harm caused by childhood victimisation. Importantly, interventions must move beyond behavioural management to therapeutic and family‐oriented models. The findings also highlight the importance of specialist training for practitioners working with adolescents who use violence to better understand the complex interplay between disability, mental health, and past experiences of child abuse. Finally, this research supports the need for dedicated responses to AFV across Australian jurisdictions, integrated into broader efforts to end domestic, family and sexual violence in Australia. This requires substantive cross‐sector collaboration between education and family violence services, child and family services, child protection, disability, mental health and youth justice. Further research, particularly longitudinal studies, would also help to continue to build the evidence base on causal pathways and to inform effective prevention and early intervention strategies.

## Ethics Statement

Ethical approval for the project was granted by the Monash University Human Research Ethics Committee (project ID: 27269).

## Conflicts of Interest

The authors declare no conflicts of interest.

## Data Availability

The data that support the findings of this study are available on request from the corresponding author, subject to approval by all authors and the funding body. The data are not publicly available due to privacy or ethical restrictions.

## Endnotes

^1^
Notably respondents assigned female at birth make up 67.35% of the sample.

^2^
The interaction model was removed for clarity. See model with interaction in Appendix A.

^3^
Natural direct and indirect effects through moderation can also be ruled out. See Appendix A.

## Appendix A

Moderation analysis: Interaction between *poor mental health* and *victimisation of violence* (*N* = 4724; F(9, 4714) = 123.14, *R* ^2^ = 0.1903, Adj. *R* ^2^ = 0.1888, RMSE = 0.3626).

**Table:**

Predictor	B	SE	t	*p*	95% CI lower	95% CI upper
Poor mental health	0.0569	0.0182	3.12	0.002	0.0211	0.0926
Victimisation of violence	0.3703	0.0141	26.26	< 0.001	0.3426	0.3979
Poor mental health × Victimisation of violence	−0.0236	0.0266	−0.89	0.375	−0.0758	0.0286
Sex	0.0085	0.0118	0.72	0.470	−0.0146	0.0316
Gender normativity (GENDER cat2)	−0.0002	0.0288	−0.01	0.993	−0.0568	0.0563
Culture (NESB)	−0.0306	0.0176	−1.73	0.083	−0.0652	0.0040
ATSI	0.0011	0.0008	1.39	0.165	−0.0004	0.0026
Education	−0.0007	0.0045	−0.15	0.877	−0.0095	0.0081
Country of birth (COB2)	−0.0002	0.0018	−0.13	0.897	−0.0038	0.0033
Constant	0.1127	0.0441	2.56	0.011	0.0262	0.1991

*Note:* The interaction term (Poor mental health × Victimisation of violence) was not significant, indicating that the effect of childhood abuse on AFV via poor mental health does not differ by levels of childhood abuse.
